# Primary Sensorimotor Cortex Drives the Common Cortical Network for Gamma Synchronization in Voluntary Hand Movements

**DOI:** 10.3389/fnhum.2018.00130

**Published:** 2018-04-06

**Authors:** Gertrúd Tamás, Venkata C. Chirumamilla, Abdul R. Anwar, Jan Raethjen, Günther Deuschl, Sergiu Groppa, Muthuraman Muthuraman

**Affiliations:** ^1^Department of Neurology, Semmelweis University, Budapest, Hungary; ^2^Section of Movement Disorders and Neurostimulation, Biomedical Statistics and Multimodal Signal Processing Unit, Department of Neurology, Focus Program Translational Neuroscience (FTN), University Medical Center of the Johannes Gutenberg University Mainz, Mainz, Germany; ^3^Department of Neurology, Christian-Albrechts-University, Kiel, Germany; ^4^Biomedical Engineering Centre, University of Engineering and Technology, Lahore, Pakistan

**Keywords:** gamma synchronization, network, connectivity, directionality, hand movements

## Abstract

**Background:** Gamma synchronization (GS) may promote the processing between functionally related cortico-subcortical neural populations. Our aim was to identify the sources of GS and to analyze the direction of information flow in cerebral networks at the beginning of phasic movements, and during medium-strength isometric contraction of the hand.

**Methods:** We measured 64-channel electroencephalography in 11 healthy volunteers (age: 25 ± 8 years; four females); surface electromyography detected the movements of the dominant hand. In Task 1, subjects kept a constant medium-strength contraction of the first dorsal interosseus muscle, and performed a superimposed repetitive voluntary self-paced brisk squeeze of an object. In Task 2, brisk, and in Task 3, constant contractions were performed. Time-frequency analysis of the EEG signal was performed with the multitaper method. GS sources were identified in five frequency bands (30–49, 51–75, 76–99, 101–125, and 126–149 Hz) with beamformer inverse solution dynamic imaging of coherent sources. The direction of information flow was estimated by renormalized partial directed coherence for each frequency band. The data-driven surrogate test, and the time reversal technique were performed to identify significant connections.

**Results:** In all tasks, we depicted the first three common sources for the studied frequency bands that were as follows: contralateral primary sensorimotor cortex (S1M1), dorsolateral prefrontal cortex (dPFC) and supplementary motor cortex (SMA). GS was detected in narrower low- (∼30–60 Hz) and high-frequency bands (>51–60 Hz) in the contralateral thalamus and ipsilateral cerebellum in all three tasks. The contralateral posterior parietal cortex was activated only in Task 1. In every task, S1M1 had efferent information flow to the SMA and the dPFC while dPFC had no detected afferent connections to the network in the gamma range. Cortical-subcortical information flow captured by the GS was dynamically variable in the narrower frequency bands for the studied movements.

**Conclusion:** A distinct cortical network was identified for GS in voluntary hand movement tasks. Our study revealed that S1M1 modulated the activity of interconnected cortical areas through GS, while subcortical structures modulated the motor network dynamically, and specifically for the studied movement program.

## Introduction

Synchronized gamma oscillatory activity was associated with neural coding (Gray, 1999). In the cortex, it is supposed to originate from the interaction of excitation and inhibition in local neural circuits (Buzsáki and Wang, 2012) to which inhibitory interneurons may contribute the most (Suffczynski et al., 2014). Gamma activity may promote information processing in task-specific neuron networks (Buzsáki and Schomburg, 2015).

Gamma synchronization (GS) may reflect a prokinetic state in the motor network (Lalo et al., 2008); its role in the fine sensorimotor control was widely examined with different modalities. Synchronization of the 30–100 Hz frequency gamma range could be detected at the beginning and at the end of a simple movement above the contralateral primary sensorimotor cortex in several studies with electrocorticography (ECoG) (Crone et al., 1998; Pfurtscheller et al., 2003; Ball et al., 2008), electroencephalography (EEG) (Ball et al., 2008), magnetoencephalography (MEG) (Waldert et al., 2008; Wilson et al., 2010; Huo et al., 2011) and stereoelectroencephalography (Szurhaj et al., 2006). A low (∼30–60 Hz) and high frequency component (>50–60 Hz) of GS was distinguished (Crone et al., 1998; Demandt et al., 2012) in the motor system. High frequency GS is transient and generated during movement onset. Low frequency GS lasts longer and follows the beginning of the movement after 200–500 ms (Crone et al., 1998; Szurhaj et al., 2003). Such gamma activity was also identified in the cortex, which was coherent with the Piper rhythm, a low gamma range electromyography (EMG) oscillation of submaximal and maximal isometric muscle contractions (Conway et al., 1995; Brown et al., 1998; Gross et al., 2005). The timing of GS, and its absence during passive movement, supports its role in the latter stage of the motor planning processes (Muthukumaraswamy, 2010). Although broad-band GS was also detected in earlier EEG and ECoG studies in the motor system (Crone et al., 1998; Ball et al., 2008), it appeared in separate narrow frequency bands in several electrophysiological measurements (Pfurtscheller et al., 2003; Cheyne et al., 2008; Darvas et al., 2010; Crone et al., 2011). It is hypothesized that these separate gamma activities may belong to different cortical modules (Pfurtscheller et al., 2003).

In the alpha/mu and beta frequency ranges, desynchronization accompanies the gamma synchronization in the motor system. Alpha and beta synchronization appears at the end of the movement and may represent cortical inhibition (Pfurtscheller and Lopes da Silva, 1999). Beta activity has denser networks and is more engaged in the coordination of sensorimotor information processing than the mu rhythm (Athanasiou et al., 2016). Gamma oscillations are coordinated by the slower rhythms locally or across anatomical regions (Buzsáki and Wang, 2012). The frequency bands having dynamic cross-frequency coupling are specific to particular brain regions (Canolty and Knight, 2010; Buzsáki and Wang, 2012). In the sensorimotor system, high gamma (80–150 Hz) amplitude was coupled with the alpha phase in hand movements (Yanagisawa et al., 2012).

In the present study, we measured the low and high frequency GS related to the onset of phasic hand movements and a medium-strength isometric contraction in healthy subjects with EEG. Our aim was to analyze the sources of the narrow-frequency band GS and the directionality of connections within the sub-networks in three different hand movements.

During the source analysis, our primary goal is to estimate the gamma activity at a source point while avoiding the crosstalk from other regions so that their effect is as little as possible on the estimate of the region of interest. Thus focusing at a particular source point is known as beamforming. In this case the source point is directed toward the field of reconstruction of neuronal sources generating EEG and MEG data in order to increases the sensitivity of signals coming from a region of interest inside the brain (Van Drongelen et al., 1996; Van Veen et al., 1997; Sekihara et al., 2001). These techniques are based on spatial adaptive filters that allow the estimating of the amount of activity at any given location in the brain. Beamforming comes in different approaches depending on which domain the estimates are performed in: the linearly constrained minimum variance (LCMV) (Van Veen et al., 1997), and the synthetic-aperture magnetometry (SAM) (Vrba and Robinson, 2001) that rely on time-domain estimates, whereas dynamic imaging of coherent sources (DICS) (Gross et al., 2001) relies on frequency-domain estimates. The DICS algorithm has been used for many years in motor research, both for MEG (Timmermann et al., 2003; Schnitzler et al., 2006) and EEG signals (Muthuraman et al., 2014; Pedrosa et al., 2017). There is now gaining interest in using it in other fields, such as in epilepsy (Moeller et al., 2012; Elshoff et al., 2013).

## Materials and Methods

**FIGURE 1:**
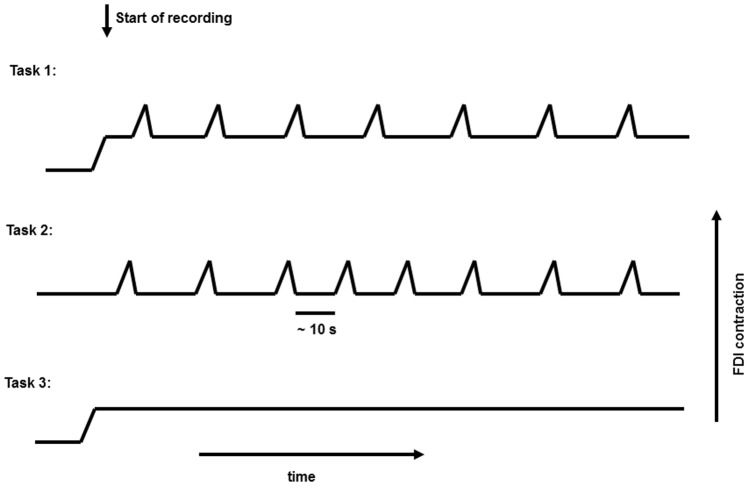
Representative figure for the three different tasks performed.

In this study, 11 healthy subjects (age: 25 ± 8 years; four females) were recruited. All gave written informed consent. The study was approved by the Ethics Committee, Medical Faculty, University of Kiel. The subjects were seated comfortably in an armchair with their forearms supported, and their hands hanging freely from the armrests. They kept their eyes open during all tasks, while focusing on one point. In the first task (Task 1), subjects kept a constant medium-strength contraction of the first dorsal interosseous (FDI) muscle (holding a 1000 g weight); and superimposed on this contraction they performed a repetitive voluntary self-paced brisk squeeze of the object (combined movement) approximately every 10 s. In the second task (Task 2), the hands were supported, and the subjects executed only the brisk contraction with complete rest in between. In the third task (Task 3), only the medium-strength constant contraction was performed (**Figure 1**). For detection of muscle activity, and marking the beginning and the end of brisk voluntary movement, a bipolar surface EMG electrode was placed above the FDI muscle.

The average duration of the brisk movements in all subjects was compared in the first and second tasks with the Wilcoxon matched-pairs test. The average EMG activity of isometric contractions in Tasks 1 and 3 were compared, as well as the average EMG activity of brisk movements and average EMG activity of isometric contractions measured in Task 1, with Wilcoxon matched-pairs test. EMG results are published in an earlier paper (Muthuraman et al., 2012c), and are briefly summarized here in the results section.

EMG was recorded in parallel with a standard 64-channel EEG recording system (Brain Products Co., Munich, Germany) using a linked mastoid reference. A standard EEG cap was used with electrodes positioned according to the extended 10–20 system. EEG and EMG were band-pass filtered (EMG 30–200 Hz; EEG 0.05–300 Hz), and sampled at 1000 Hz. In addition, a notch filter was used to filter out the 50, 100, and 150 Hz activity. Data were stored in a computer and analyzed off-line. The EMG was full-wave rectified (Muthuraman et al., 2010a).

The rectified EMG signal was used to identify the beginning of the movements; we flagged the EMG and EEG signal at the beginning of the movements with “on” markers. We created 8-s-long EEG segments, 4s before and 4s after the “on” marker position as the time 0. Ocular artifacts were controlled visually at the F1, Fz, and F2 EEG channels. Segments with visible artifacts were manually rejected. Trials were only selected when the beginning of the movement could be clearly defined. In each phasic task (Task 1 and 2), 35 ± 5 segments were utilized for further analysis.

We have used the average reference scheme for our entire scalp and source analyses (Nunez, 2010). The scalp time-frequency analyses were rechecked with the zero reference scheme. Its principle is to take the reference of the scalp EEG to a point at infinity, which is far from all the possible neural sources. This method is called the reference electrode standardization technique (REST) (Yao, 2001).

### Time Frequency Analysis

The dynamics of signals in the time and frequency domains were computed with the multitaper method (Mitra and Pesaran, 1999). In this method the spectrum is estimated by multiplying the data *x(t)* with *K* different windows (i.e., tapers). The complete description of the method is explained elsewhere (Muthuraman et al., 2010a). The time step was 50 ms with overlapping windows of 1000 ms, providing an approximate time resolution of 50 ms and an approximate frequency resolution of 1 Hz. After calculating the absolute power spectra, we estimated the relative event-related power changes for each 1 Hz wide frequency band; the reference interval was chosen from -4 s to -3 s. We averaged the relative data from 0 to 2.5 s in five frequency bands (30–49, 51–75, 76–99, 101–125, and 126–149 Hz) across subjects. In Task 3 (isometric contractions) we divided the absolute data set into 2.5 s segments that were used for further processing.

### Source Analysis

The dynamic imaging of coherent sources (DICS) (Gross and Ioannides, 1999; Gross et al., 2001) was used to identify the sources responsible for the five fixed gamma frequency bands. The EEG signal was average referenced before the source analyses. The complete description of the methods is described elsewhere (Muthuraman et al., 2012a; Michels et al., 2013). In short, to locate the origin of a specific EEG activity seen on the scalp, the forward and inverse problems need to be solved. The forward problem is the computation of the scalp potentials for a set of neural current sources. Estimating the so-called lead-field matrix with specified models for the brain usually solves it. In this study, the more complex five-concentric-spheres model was used to create the volume conductor model with standard T1 magnetic resonance images (Zhang, 1995). The open source software used here was Fieldtrip (Oostenveld et al., 2011). The lead-field matrix (LFM) needed to be calculated to map the current dipole in the human brain to the voltages on the scalp. It was estimated using the boundary-element method (BEM) (Fuchs et al., 2002).

The inverse problem is the quantitative estimation of the properties of the neural current sources underlying the neural activity. The power at any given location in the brain can be computed using a linear transformation, which in this case is the spatial filter (Van Veen et al., 1997). The linearly constrained minimum variance (LMCV) spatial filter was used in this study, which relates the underlying neural activity to the electromagnetic field on the surface.

**FIGURE 2:**
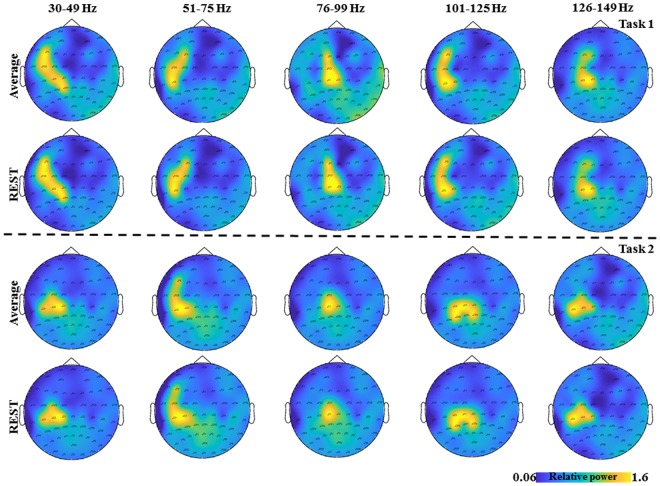
The topological plots of the grand average GS from all the subjects for the five frequency bands separately (30–49, 51–75, 76–99, 101–125, and 126–149 Hz) and time interval (0–2.5 s) are depicted. The first row shows the topological plots of the Task 1 for common average reference and the second row shows the plots for the zero reference scheme. The third row shows the topological plots of the Task 2 for common average reference and the fourth row shows the plots for the zero reference scheme. The color bar represents the relative power in the interval 0–2.5 s.

**FIGURE 3:**
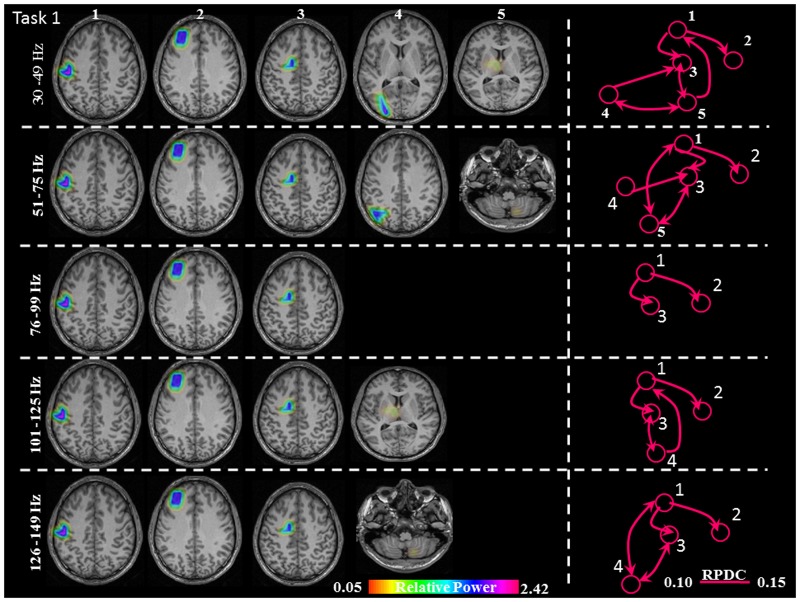
Sources of relative gamma synchronization at the beginning of the combined Task 1 and directionality of information flow within the network in the five frequency bands. The color bar represents the relative power. The individual maps of power were spatially normalized and interpolated on standard T1-weighted MRI scans with 1 mm spacing in MNI space. 1: primary sensorimotor cortex (S1M1); 2: dorsolateral prefrontal cortex (dPFC); 3: supplementary motor area (SMA); 4/5: posterior parietal cortex (PPC)/thalamus (TH)/cerebellum (CER); RPDC, renormalized partial directed coherence.

**FIGURE 4:**
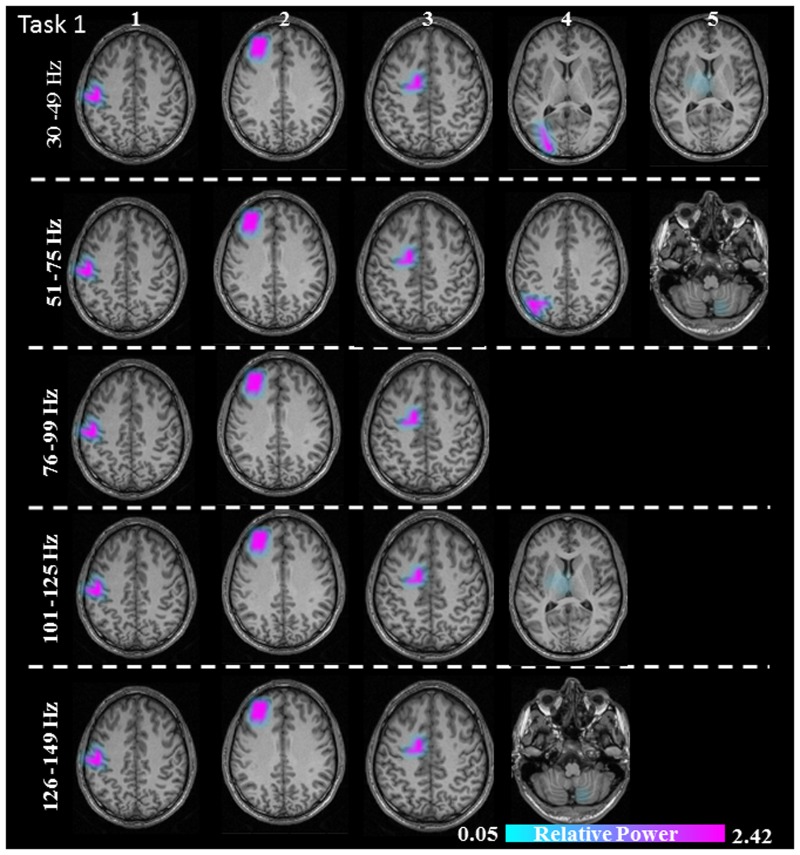
Sources of relative gamma synchronization at the beginning of the combined Task 1 with the REST reference scheme in the five frequency bands. The color bar represents the relative power. The individual maps of power were spatially normalized and interpolated on standard T1-weighted MRI scans with 1 mm spacing in MNI space. 1: primary sensorimotor cortex (S1M1); 2: dorsolateral prefrontal cortex (dPFC); 3: supplementary motor area (SMA); 4/5: posterior parietal cortex (PPC)/thalamus (TH)/cerebellum (CER).

**FIGURE 5:**
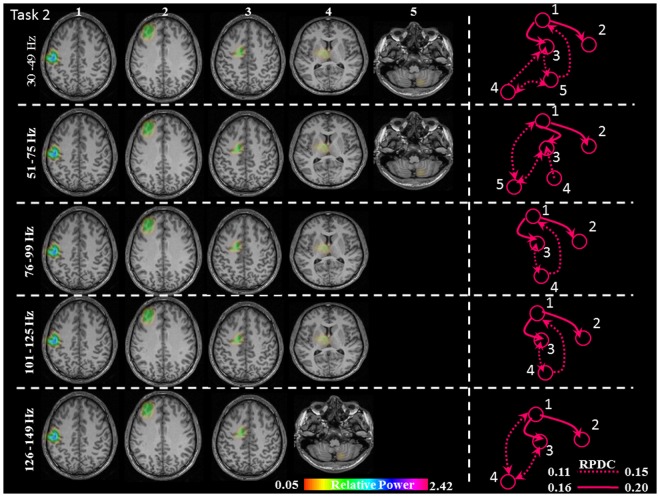
Relative gamma synchronization at the onset of the brisk pressing, Task 2. Results of the source and directionality analysis in the five frequency bands. The individual maps of power were spatially normalized and interpolated on standard T1-weighted MRI scans with 1 mm spacing in MNI space. The color bar represents the relative power. 1: primary sensorimotor cortex (S1M1); 2: dorsolateral prefrontal cortex (dPFC); 3: supplementary motor area (SMA); 4/5: thalamus (TH)/cerebellum (CER); RPDC, renormalized partial directed coherence.

**FIGURE 6:**
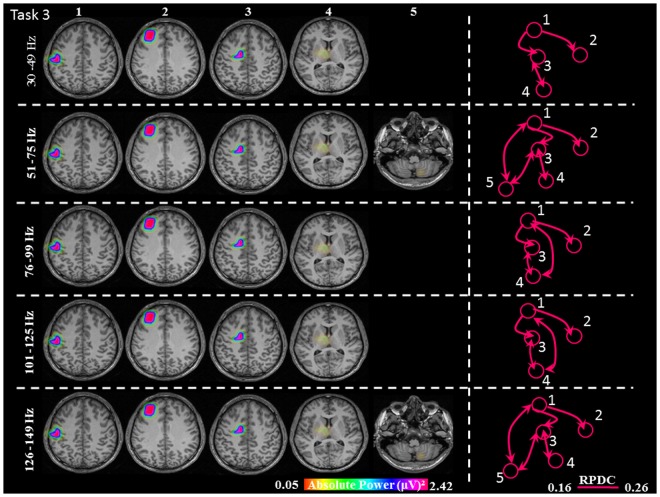
Absolute gamma activity during the continuous Task 3. Estimated sources and direction of information flow in the five frequency bands. The individual maps of power were spatially normalized and interpolated on standard T1-weighted MRI scans with 1 mm spacing in MNI space. The color bar represents the relative power. 1: primary sensorimotor cortex (S1M1); 2: dorsolateral prefrontal cortex (dPFC); 3: supplementary motor area (SMA); 4/5: thalamus (TH)/cerebellum (CER); RPDC, renormalized partial directed coherence.

The source analysis was carried out based on the time lock analysis within the time interval of the GS between 0 and 2.5 s in Task 1 and Task 2. We analyzed each 2.5 s segment as a whole in Task 3. We identified the source of the strongest power in the frequency bands in the first run of the source analysis and then considered this source as noise for the next run to identify further sources. The individual maps of power were spatially normalized and interpolated on standard T1-weighted MRI scans with 1 mm spacing in MNI space. In a further analysis, all initial power estimates of the individual source voxels were combined to get a pooled power estimate for each source separately. This can be done by computing the individual second order spectra using a weighting scheme and estimating the power to obtain the pooled power spectra of all the significant individual voxels (Rosenberg et al., 1989; Amjad et al., 1997). Later, the pooled source time series were used for the connectivity analyses separately at each of the five gamma frequency bands for all subjects. The source analysis was carried out on an individual basis, and then followed up by a grand average analysis for **Figures 2**–**6**. The whole description of the forward solution for the five concentric sphere models (Muthuraman et al., 2012b) and the boundary element method (Muthuraman et al., 2010b). For the inverse solution the spatial filter is described in our previous paper with derivations (Muthuraman et al., 2008).

### Connectivity Analysis

Renormalized partial directed coherence (RPDC) (Schelter et al., 2009) is a technique performed in the frequency domain to detect causal influences in multivariate stochastic systems and provides information on the direction of information flow between the source signals. The complete description of this method is described elsewhere (Muthuraman et al., 2012a; Michels et al., 2013). Briefly, the multivariate model was used to model the source signals, which uses an autoregressive process to obtain the coefficients of the signals in the desired frequency band of interest. In order to obtain these coefficients, the optimal model order for the corresponding signal needed to be chosen by minimizing the Akaike Information Criterion (AIC) (Akaike, 1974; Ding et al., 2000). After estimating the RPDC values, the significance level was calculated from the applied data using a bootstrapping method (Kaminski et al., 2001). The open source Matlab package autoregressive fit (ARFIT) (Neumaier and Schneider, 2001; Schneider and Neumaier, 2001) was used for estimating the autoregressive coefficients from the spatially filtered source signals. We applied the time reversal technique TRT (Haufe et al., 2013) as a second significance test on the connections already identified by RPDC using the bootstrapping, which is a data-driven surrogate significance test.

### Statistical Analysis

The inter-individual differences in the source locations within each task (*n* = 11, *p* < 0.05) were tested using a non-parametric Kruskal–Wallis test. For this, we estimated the number of activated voxels for the first three common sources in all the frequency bands. Secondly, we took the MNI coordinates of the first three common sources in all the frequency bands, and estimated the Euclidean distance among the subjects using a non-parametrical Kruskal–Wallis test. The statistical significance of the sources (*n* = 11, *p* < 0.05) was tested by a within-subject surrogate analysis. A Monte-Carlo test of 100 random permutations was carried out and the *p*-values were calculated for each permutation (Maris and Oostenveld, 2007; Maris et al., 2007); the 99th percentile *p*-value was taken as the significance level for each subject (Muthuraman et al., 2012a). The RPDC values between the source signals were tested for significance using the multifactorial ANOVA; the within-subject factors were the interactions of the source signals the number of connections and the between subject factors were the tasks (*n* = 3) and the frequency bands (*n* = 5). The Bonferroni correction was performed for all *post hoc* tests.

We analyzed whether the power of the source and the connectivity values depend on movement complexity and gamma band frequency in the cortical motor network. We performed ANOVA for repeated measures, since sets of grouped data had normal distribution according to the Kolmogorov–Smirnov test. The following within-subject factors were determined for the relative GS at the beginning of the movement in Task 1 and Task 2: task (Task 1 and Task 2), cortex (S1M1, SMA, dPFC) and frequency (five ranges). Cortex and frequency within-subject factors were analyzed for the absolute power values in Task 1; and task, cortical connections and frequency within-subject factors for the connectivity values measured in Task 1–3. We used the Bonferroni test for *post hoc* comparisons. The level of significance was set to *p* < 0.05 for all statistical analyses.

## Results

The average duration of the brisk contraction was longer in Task 1 (0.43 ± 0.06 s) than in Task 2 (0.4 ± 0.07s, *p* = 0.03). EMG activity of the constant isometric contraction was higher in Task 1 than in Task 3 (346.1 ± 321.01 μV and 297.9 ± 287.84 μV, respectively; *p* = 0.007). As expected, EMG activity increased significantly during the brisk movements as compared to the constant isometric contraction in Task 1 (*p* = 0.002). In Task 2, there was no EMG activity between the brisk movements (Muthuraman et al., 2012c). In order to demonstrate the results on the time frequency analyses from Task 1 (**Figure 2** first two rows) and Task 2 (**Figure 2** last two rows) the grand average from all the subjects for the five frequency bands are shown separately (30–49, 51–75, 76–99, 101–125, 126–149 Hz) and time interval (0–2.5 s) are shown in **Figure 2**, for two different reference schemes, namely, common average and zero reference schemes (Dong et al., 2017; Yao, 2017).

### Source Analysis

The sources discussed below were all significant over all subjects, in each task separately (Task 1: *p* = 0.004; Task 2: *p* = 0.003; Task 3: *p* = 0.001). In Task 1 (constant isometric contractions combined with brisk contractions), the network of sources involved the contralateral S1M1, the dorsolateral prefrontal cortex (dPFC), and the supplementary motor area (SMA) for all five frequency bands, namely 30–49, 51–75, 76–99, 101–125, and 126–149 Hz. Additionally, the contralateral posterior parietal cortex (PPC) was activated in the low gamma frequency band (30–75 Hz). Thalamus gamma activity could be detected in the 30–49 Hz and the 101–125 Hz bands. GS appeared in the ipsilateral cerebellum (C) in the 51–75 Hz and the 126–149 Hz frequency ranges (**Figure 3**). We have reanalyzed the source data in Task 1 with the REST reference scheme and show the results in **Figure 4**. This reference scheme did not make any significant peak voxel activation difference for the first source (*p* = 0.45) or relative power difference (*p* = 0.67) to the results of the common average scheme.

In Task 2 (brisk contractions), the network of sources involved the contralateral S1M1, dPFC and SMA for all five frequency bands. Except for the frequency band 126–149 Hz, all of the other frequency bands had a contralateral thalamus source; the frequency bands 30–49, 51–75, and 126–149 Hz had an ipsilateral cerebellum source (**Figure 5**).

During the constant isometric contractions in Task 3, the delimited network showed significant sources in the contralateral S1M1, dPFC, SMA and the contralateral thalamus source for all five frequency bands. Additionally, the frequency bands 51–75 Hz, and 126–149 Hz had an ipsilateral cerebellum source (**Figure 6**).

**FIGURE 7:**
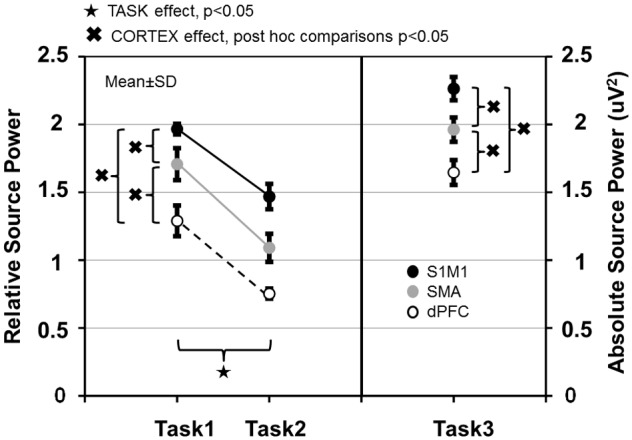
Relative source power values of gamma synchronization in Task 1–2 and absolute source power values in Task 3 measured in the primary sensorimotor cortex (S1M1), supplementary motor area (SMA) and dorsolateral prefrontal cortex (dPFC) areas. Values are averaged across frequencies.

In the three involved cortical areas (S1M1, dPFC, SMA) source power was larger in Task 1 than in Task 2 (**Figure 7**). Comparing the cortical areas, source power was the largest in S1M1; it was smaller in the SMA, and even smaller in the dPFC in all three tasks (**Figure 7**). The results of the statistical analyses are presented in **Table 1**.

**Table 1:** Statistical analysis of the gamma source power and the connectivity values measured in the motor cortical areas in the three tasks.

ANOVA effect		Bonferroni *post hoc* comparisons, significant differences
**Relative gamma source power in Task 1 and Task 2**		
All	*F*_1,10_ = 39666.05; *p* < 0.01	
Task	*F*_1,10_ = 2595.02; *p* < 0.01	*p*_*Task*1-*Task*2_ < 0.01
Cortex	*F*_2,20_ = 2731.53; *p* < 0.01	In every comparison *p* < 0.01
Frequency	*F*_4,40_ = 0.94; *p* = 0.45	
Task × Cortex	*F*_2,20_ = 14.1; *p* < 0.01	In every comparison *p* < 0.01
Task × Frequency	*F*_4,40_ = 1.4; *p* = 0.25	
Cortex × Frequency	*F*_8,80_ = 1.47; *p* = 0.182	
Task × Cortex × Frequency	*F*_8,80_ = 0.7; *p* = 0.692	
**Absolute gamma source power in Task 3**		
All	*F*_1,10_ = 132676.1; *p* < 0.01	
Cortex	*F*_2,20_ = 752.8; *p* < 0.01	In every comparison *p* < 0.01
Frequency	*F*_4,40_ = 3.8; *p* = 0.01	*p*_(76-100 *Hz*)-(126-150 *Hz*)_ < 0.01
Cortex × Frequency	*F*_8,80_ = 1.3; *p* = 0.253	
**Connectivity values in the gamma bands**		
All	*F*_1,10_ = 128829.5; *p* < 0.01	
Task	*F*_2,20_ = 3773.7; *p* < 0.01	In every comparison *p* < 0.01
Cortex	*F*_1,10_ = 0.3; *p* = 0.59	
Frequency	*F*_4,40_ = 0.9; *p* = 0.48	
Task × Cortex	*F*_2,20_ = 185.8; *p* < 0.01	In every comparison *p* < 0.01, except: *p*_*Task*2, *S*1*M*1-dPFCvs. *Task*2, *S*1*M*1-*SMA*_ = 0.99
Task × Frequency	*F*_8,80_ = 2.4; *p* = 0.02	Connectivity values of the different frequency bands were similar in each task (*p* > 0.05). Values in the three tasks were different (*p* < 0.01).
Cortex × Frequency	*F*_4,40_ = 0.3; *p* = 0.86	
Task × Cortex × Frequency	*F*_8,80_ = 0.8; *p* = 0.5871	


The number of activated voxels in the three identified first common three sources was not significantly different in the tasks (*p* > 0.05), or in the five frequency bands (*p* > 0.05). The voxel coordinates (x, y, z) with the maximum amplitude in the first common three sources in all three tasks and the five frequency bands were the same in 8 of 11 subjects in Task 1 (in the other three subjects, the differences in the most active voxel coordinates were: min*_x_*: 0, max*_x_*: 3, interquartile range: 2; min*_y_*: 0, max*_y_*: 3, interquartile range: 2; min*_z_*: 0, max*_z_*: 0). These coordinates were the same in 7 of 11 subjects in Task 2 (the differences in the four other subjects were: min*_x_*: 0, max*_x_*: 3, interquartile range: 2; min*_y_*: 0, max*_y_*: 6, interquartile range: 4; min*_z_*: 0, max*_z_*: 0). The coordinates were the same in 8 of 11 subjects in Task 3 (in 3 subjects, the differences in the most active voxel coordinates were: min*_x_*: 0, max*_x_*: 3, interquartile range: 2; min*_y_*: 0, max*_y_*: 3, interquartile range: 2; min*_z_*: 0, max*_z_*: 0). There were no significant inter-individual differences in the relative voxel coordinates of the sources estimated for the five frequency bands in the three tasks (*p* > 0.05).

### Connectivity Analyses

**FIGURE 8:**
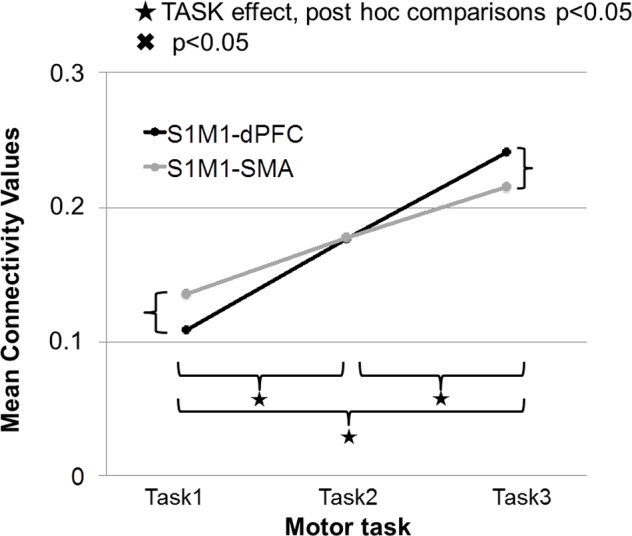
Strength of connectivity between primary sensorimotor cortex (S1M1) and supplementary motor area (SMA), and between S1M1 and dPFC in the three movement tasks. Values are averaged across frequencies.

In this section, we discuss only the significant connections between the identified sources for the different gamma frequency bands and specific tasks. The strength of connectivity between S1M1 and dPFC and between S1M1 and SMA was the largest in Task 3; it was smaller in Task 2 and even smaller in Task 1 (**Figure 8**). The TASK effects, and its post-hoc comparisons, were all significant (**Table 1**). Connectivity was similar in the five frequency bands (FREQUENCY effect: *p* > 0.05; **Table 1**).

In Task 1, the effective connectivity was significantly stronger between S1M1 and SMA than between S1M1 and dPFC (**Figure 8** and **Table 1**). The information flow was directed from S1M1 to dPFC, and from S1M1 to SMA. Additionally, in the frequency band 30–49 Hz, the information flow was from posterior parietal to the SMA, and had bi-directional information flows with the thalamus. The information flow was from thalamus to the S1M1, and had bi-directional flow with the SMA in the 31–49 Hz and 101–125 Hz bands. The frequency band 51–75 Hz showed information flow from PPC to SMA. Bi-directional flow between the C and S1M1, and between C and SMA was significant in the 51–75 Hz, and 126–149 Hz frequency bands (**Figure 3**).

In Task 2, there were the same three common sources in all gamma bands as in Task 1: S1M1, dPFC and SMA; the information flow was directed from S1M1 to dPFC and from S1M1 to SMA (**Figure 5**). The strength of connectivity in these two pathways was not significantly different (**Figure 8** and **Table 1**). Uni- (in the 30–75 Hz band) or bi-directional (in the 76–99 and 101–125 Hz bands) flow could be calculated between thalamus and SMA. The information flow was directed from the thalamus to S1M1 in the 76–99 and 101–125 Hz frequency bands. Except for the frequency bands 76–99 and 101–125 Hz, all the other frequency bands showed bi-directional flow between C and the SMA, similar to the findings in Task 1. The C sent information to the S1M1 in the 30–49 Hz band; this connection was bi-directional in the 51–75 and 126–149 Hz bands. However, the mean sub-cortical to cortical connections was significantly weaker (*p* = 0.005) than the main cortico-cortical connections.

In Task 3, the cortical connections were unidirectional from the S1M1 to the dPFC and the SMA as in Task 1 and Task 2. The connectivity was significantly stronger between S1M1 and dPFC than between S1M1 and SMA (**Figure 8** and **Table 1**). In the frequency bands 51–75 and 126–149 Hz we found bi-directional connections between the C and S1M1, and between the C and the SMA. In all frequency bands, we estimated bi-directional information flow between the thalamus and SMA (**Figure 6**), similar to the findings in some frequency bands in Task 1.

## Discussion

In this study, we found a common neural network for the narrow frequency band GS at the beginning of two different phasic movements, and during a medium-strength isometric contraction. We confirm that GS arises from functionally connected cortical and subcortical neuron populations, and can dynamically change in different tasks. This is the first study using directionality analysis within the motor networks for parallel GS.

The main findings of the study:

(1) The common network involved the S1M1, SMA, dPFC, thalamus and the cerebellum. PPC was also activated in the combined Task 1.
(2) Connections between the three cortical sources could be observed in every analyzed gamma band, while subcortico-cortical connections were represented in various gamma bands in the tasks.
(3) S1M1 set the activity of other cortical sources in the gamma band.
(4) The dPFC had no afferent input to the gamma network.
(5) The source power was highest in S1M1, and then sequentially less in the SMA and in the dPFC, in every task.
(6) The source power at the beginning of the combined movement was higher than at the beginning of the simple movement in every studied cortex area.
(7) The beginning of the combined movement was accompanied by the lowest connectivity between cortical sources. Strength of connectivity between S1M1 and SMA, and between S1M1 and dPFC depended on the type of movement.

We did not find significant inter-individual differences in the source analyses; this might be due to usage of standard template MRI scans, and standard electrode locations, instead of individual metrics.

We analyzed the GS at the beginning of both a simple brisk and a combined movement, and during a medium-strength isometric contraction. GS at the beginning of the movement is a principle electrophysiological phenomenon (Crone et al., 1998; Pfurtscheller et al., 2003; Ball et al., 2008). We also found low and high gamma activity in the motor network during medium-strength isometric contractions, despite it being associated with beta band phenomena in earlier studies. Cortico-muscular coherent activity in the motor cortex (Conway et al., 1995; Brown et al., 1998), and coupling activity between the primary motor and supplementary motor cortex (Herz et al., 2012), was earlier detected only in the beta band. Low gamma band cortico-muscular coherent coupling was indeed found during submaximal and maximal isometric muscle contractions (Conway et al., 1995; Brown et al., 1998; Gross et al., 2005), or during dynamic force output (Andrykiewicz et al., 2007); it was suggested that its frequency band may vary individually (Naranjo et al., 2010).

We identified the sources contralateral to the movement, except for the ipsilateral cerebellum. Our results are in agreement with previous studies that localized the GS to the contralateral perirolandic area (Crone et al., 1998; Cheyne et al., 2008; Darvas et al., 2010), and also the SMA (Ball et al., 2008; Wilson et al., 2010; Demandt et al., 2012) bilateral with contralateral preponderance (Ohara et al., 2000).

In the S1M1, several earlier studies analyzed the spatial distribution and temporal evolution of low and high GS. The low GS spreads to a wider cortical area, it is bilateral and has contralateral predominance (Ohara et al., 2000; Miller et al., 2007; Demandt et al., 2012); whereas, high GS develops spatially, and is more focal only on the contralateral side (Crone et al., 1998; Miller et al., 2007; Cheyne et al., 2008); source localization estimated its origin in either the primary motor cortex (Cheyne et al., 2008), or in the primary sensory cortex (Pfurtscheller et al., 2003; Szurhaj et al., 2003; Miller et al., 2007). We found the strongest gamma source in the S1M1 among the cortical areas in every task, as it was also demonstrated by a MEG study in children (Wilson et al., 2010). We proved that S1M1 precipitated the activity of other cortical sources in the gamma band in all three tasks. The connectivity between S1M1 and SMA was stronger than the S1M1-dPFC connectivity in the combined Task 1, which was the opposite in the constant isometric task; they were equal in the phasic task. This directional specificity may relate to the complexity of the motor task; isometric contractions may activate premotor cortex and SMA less than the combined and the brisk phasic movement, and needs continuous executive contribution (Groppa et al., 2012a). S1M1 had only afferent propagation of gamma activity from the thalamus in the combined and brisk movements. The S1M1-C information flow was bidirectional in every task, as it is expected from the action of the cerebello-thalamo-cortical, cortico-ponto-cerebellar loop (Proville et al., 2014).

We found gamma source in the contralateral SMA in all three tasks, similar to earlier studies. GS could be identified in the SMA by invasive electrophysiological studies (Pfurtscheller et al., 2003; Szurhaj et al., 2006; Miller et al., 2007). Low (30–50 Hz) and high (50–100 Hz) frequency synchronization was also demonstrated in the SMA region with EEG (Ball et al., 2008; Demandt et al., 2012; Seeber et al., 2016). In a MEG study, GS arose bilaterally in the SMA in children, with contralateral predominance (Wilson et al., 2010). In our study, SMA had a two-way connection with the thalamus and the cerebellum in every task.

Interestingly, we did not identify the premotor cortex in the core gamma network, but it had a role in the beta range information processing, as we had already published in an earlier study (Muthuraman et al., 2012c). Similarly, a previous study demonstrated a dominant role of supplementary and cingulate motor areas and not the S1M1 in alpha and beta sensorimotor networks during upper limb movement (Athanasiou et al., 2016).

We recognized that the dPFC had only afferent information flow from the motor circuit in the gamma range, which may represent executive functions during motor processing. Studies regarding dPFC reflected its engagement with working memory (D’Esposito et al., 1998), short-term memory (Majerus et al., 2010), and executive functions, such as scheduling processes in complex tasks (Smith and Jonides, 1999; Groppa et al., 2012b).

The PPC had elevated gamma activity in the combined task; it might assist the fine motor calibration. It is considered a sensory association area, and was not only associated with polysensory integration and spatial attention, but also with movement intention and planning of goal-directed actions (Andersen and Cui, 2009).

The subcortical information flow in our study was represented in specific gamma bands in the different tasks, in contrast with the cortical communication, which was identified in every gamma band. This suggests a dynamic subcortical and subcortico-cortical processing specific to the type of movement.

Our group has already identified subcortical sources using EEG in different studies (Muthuraman et al., 2012c, 2014, 2015; Chiosa et al., 2017). Subcortical sources were also already estimated by other groups using beamformer inverse solutions either from EEG or MEG data (Gross et al., 2002; Timmermann et al., 2003; Schnitzler et al., 2006; Cebolla et al., 2016).

Besides the contralateral thalamus, we recognized the ipsilateral cerebellum in the subcortical part of the gamma network. MEG previously identified a cerebellum source in the low gamma network in children (Wilson et al., 2010). We found that the cerebellum had bidirectional information flows with the S1M1 and the SMA. The cerebellum-thalamus interaction, which was bidirectional, was only found in the lowest gamma band, in Task 2. This may suggest that the cerebello-thalamic afferent pathway may act in other frequencies in the other tasks. This hypothesis is supported by animal studies, which suggested that the cerebellum may affect the coherent gamma-band activities in the primary sensory and motor cortices via the cerebello-thalamic pathway (Popa et al., 2013).

In EEG studies, the results of the scalp analyses highly depend on the used reference scheme. The common average reference is often taken as the best available reference option discussed by Nunez (2010), and revisited by Yao (2017). It was shown that its assumption is only correct for spherical volume conductor. However, the reliability of the data can be tested with two different reference schemes, as we did in this study for the scalp topological analyses in Task 1 and Task 2.

There are two major assumptions in this beamformer DICS analysis: it assumes a single dipole model, which is not linearly correlated to other dipoles. This assumption is valid if the coherence is not too strong and the signal-to-noise ratio is sufficient (Gross et al., 2001). After identifying the area of maximum power, it is projected out for finding further areas with coherent activity in the brain. Here the assumption is that the coherence between the reference and the detected activity is always 1 (Schoffelen et al., 2008). Recent usage of this type of source analyses, and connectivity analyses have been validated using different source analyses pipelines, and specifically using the common average reference for these analyses (Mahjoory et al., 2017) and also for testing of the results across different pipelines have been done (Haufe and Ewald, 2016). Furthermore, the importance of source imaging in understanding the EEG data has been emphasized (Lopes da Silva, 2013; Cohen, 2017).

Our study supports the hypothesis that there are cortical and subcortical sources of GS in functionally integrated motor networks; their participating brain areas can change with the type and complexity of the motor task. The frequency bands for the gamma activation of these brain areas may vary. Our results also highlight the dominant role of the S1M1 in gamma activity-based information processing, which supports the assumption that it is not exclusively an executive motor field (Ball et al., 1999). Dynamic connectivity changes should be further analyzed in other frequency bands to better explore the interaction of cortical and subcortical motor areas.

## Author Contributions

GT and MM were involved in conception, organization and execution of the research project; review and critique of the statistical analysis; writing of the first draft; and review and critique of the manuscript. VC and AA were involved in conception and organization of the research project and critique of the manuscript. JR was involved in organization of the research project and critique of the manuscript. GD and SG were involved in conception, organization, and execution of the research project; review and critique of the statistical analysis; writing of the first draft; and review and critique of the manuscript.

## Conflict of Interest Statement

The authors declare that the research was conducted in the absence of any commercial or financial relationships that could be construed as a potential conflict of interest.
